# Retinal Degeneration and Alzheimer’s Disease: An Evolving Link

**DOI:** 10.3390/ijms21197290

**Published:** 2020-10-02

**Authors:** Ajay Ashok, Neena Singh, Suman Chaudhary, Vindhya Bellamkonda, Alexander E Kritikos, Aaron S Wise, Neil Rana, Dallas McDonald, Rithvik Ayyagari

**Affiliations:** Department of Pathology, Case Western Reserve University, Cleveland, OH 44106, USA;

**Keywords:** glaucoma, iron, oxidative stress, inflammation, Alzheimer’s disease, reactive oxygen species, age related macular degeneration, drusen, prion protein, retinal degeneration

## Abstract

Age-related macular degeneration (AMD) and glaucoma are degenerative conditions of the retina and a significant cause of irreversible blindness in developed countries. Alzheimer’s disease (AD), the most common dementia of the elderly, is often associated with AMD and glaucoma. The cardinal features of AD include extracellular accumulation of amyloid β (Aβ) and intracellular deposits of hyper-phosphorylated tau (p-tau). Neuroinflammation and brain iron dyshomeostasis accompany Aβ and p-tau deposits and, together, lead to progressive neuronal death and dementia. The accumulation of Aβ and iron in drusen, the hallmark of AMD, and Aβ and p-tau in retinal ganglion cells (RGC), the main retinal cell type implicated in glaucoma, and accompanying inflammation suggest overlapping pathology. Visual abnormalities are prominent in AD and are believed to develop before cognitive decline. Some are caused by degeneration of the visual cortex, while others are due to RGC loss or AMD-associated retinal degeneration. Here, we review recent information on Aβ, p-tau, chronic inflammation, and iron dyshomeostasis as common pathogenic mechanisms linking the three degenerative conditions, and iron chelation as a common therapeutic option for these disorders. Additionally discussed is the role of prion protein, infamous for prion disorders, in Aβ-mediated toxicity and, paradoxically, in neuroprotection.

## 1. Introduction

Alzheimer’s disease (AD) is characterized by the gradual and progressive loss of memory and cognitive functions due to a loss of neurons in the hippocampus and, progressively, in other regions of the brain. The principal cause of neurotoxicity is the extracellular accumulation of amyloid β (Aβ), a product of abnormal processing of the amyloid precursor protein (APP), and the intracellular accumulation of tau, a microtubule stabilizing protein, as neurofibrillary tangles (NFTs) [1,2]. Inflammation and oxidative stress due to brain iron dyshomeostasis are other prominent features of AD brain pathology [3–5]. Whether these are triggered by Aβ and tau deposits or are a consequence of these changes remains unclear. The diagnosis of AD is mostly clinical, though biomarkers in the cerebrospinal fluid (CSF) provide corroborative evidence. The most reliable and frequently used biomarkers are elevated levels of Aβ_1–42_, the most toxic form of Aβ, total tau (t-tau), and hyper-phosphorylated tau (p-tau) in the CSF [6]. Vision-related changes are common in AD [7] and are summarized in Table 1. Some of the visual defects are due to degeneration of the visual cortex, while others are attributed to retinal degeneration associated with glaucoma and age-related macular degeneration (AMD) [8,9]. Thinning of the retinal nerve fiber layer (RNFL) due to the selective death of retinal ganglion cells (RGCs) has emerged as a potential diagnostic test for AD [10,11], prompting visualization of the retina by optical coherence tomography (OCT) and functional analysis with electroretinography (ERG). Though promising, these tests lack sufficient specificity and sensitivity for broad clinical applications [12–15].

Glaucomatous RGC death is typically associated with elevated intraocular pressure (IOP), the pressure exerted by the aqueous humor (AH) in the anterior segment of the eye [64,65]. Normally, AH is secreted by the ciliary epithelium and drains into venous circulation by the conventional pathway along the trabecular meshwork (TM) cells and Schlemm’s canal, and the unconventional uveoscleral pathway [66]. Elevated IOP can occur due to the alteration of extracellular matrix (ECM) proteins in the TM which become less-responsive to elevated pressure as in open-angle glaucoma, where access to the drainage pathway is blocked as in closed-angle glaucoma, or where the cause of RGC death is not clear as in normal-tension glaucoma [67,68]. It is believed that chronic elevation of IOP leads to the accumulation of Aβ and p-tau in RGCs, resulting in their loss. This leads to thinning of the RNFL and optic nerve atrophy, ultimately leading to permanent blindness because of the failure to transmit visual stimuli to the brain [7,8]. However, it is difficult to distinguish whether visual defects due to RGC cell loss appear earlier than those due to neuronal loss in the occipital cortex, information critical for the early diagnosis of AD. A recent meta-analysis of 25 studies with 887 cases of AD, 216 cases of mild cognitive impairment, and 864 healthy controls showed a positive correlation between reduced RNFL thickness and confirmed cases of AD, linking RGC death to AD brain pathology [69]. Follow-up studies either confirmed or failed to reproduce some of these observations, leaving the matter unsettled. However, the presence of AD-associated RGC death with normal or low IOP suggests a complex pathology that requires further exploration [7–9,11,64,70,71].

Retinal degeneration associated with AMD shares several features with AD, including extracellular deposits of Aβ, chronic inflammation, and oxidative stress, the latter partly attributed to the accumulation of iron [72–76]. AMD is typically associated with the accumulation of drusen between the retinal pigment epithelial (RPE) cell layer that forms the outer blood-retinal barrier and the Bruch’s membrane (BM), resulting in dysfunction of the barrier and death of photoreceptor cells. Among the various denatured proteins that comprise drusen, Aβ is prominent, suggesting a link with AD [77]. In addition, inflammation, the accumulation of iron, and oxidative stress play a central role in disease progression [74]. In a recent study of ~800 cases of AD, a significant positive correlation was noted with the established diagnosis of AMD and recent diagnosis of glaucoma [78], linking the three conditions.

**Figure 1.**
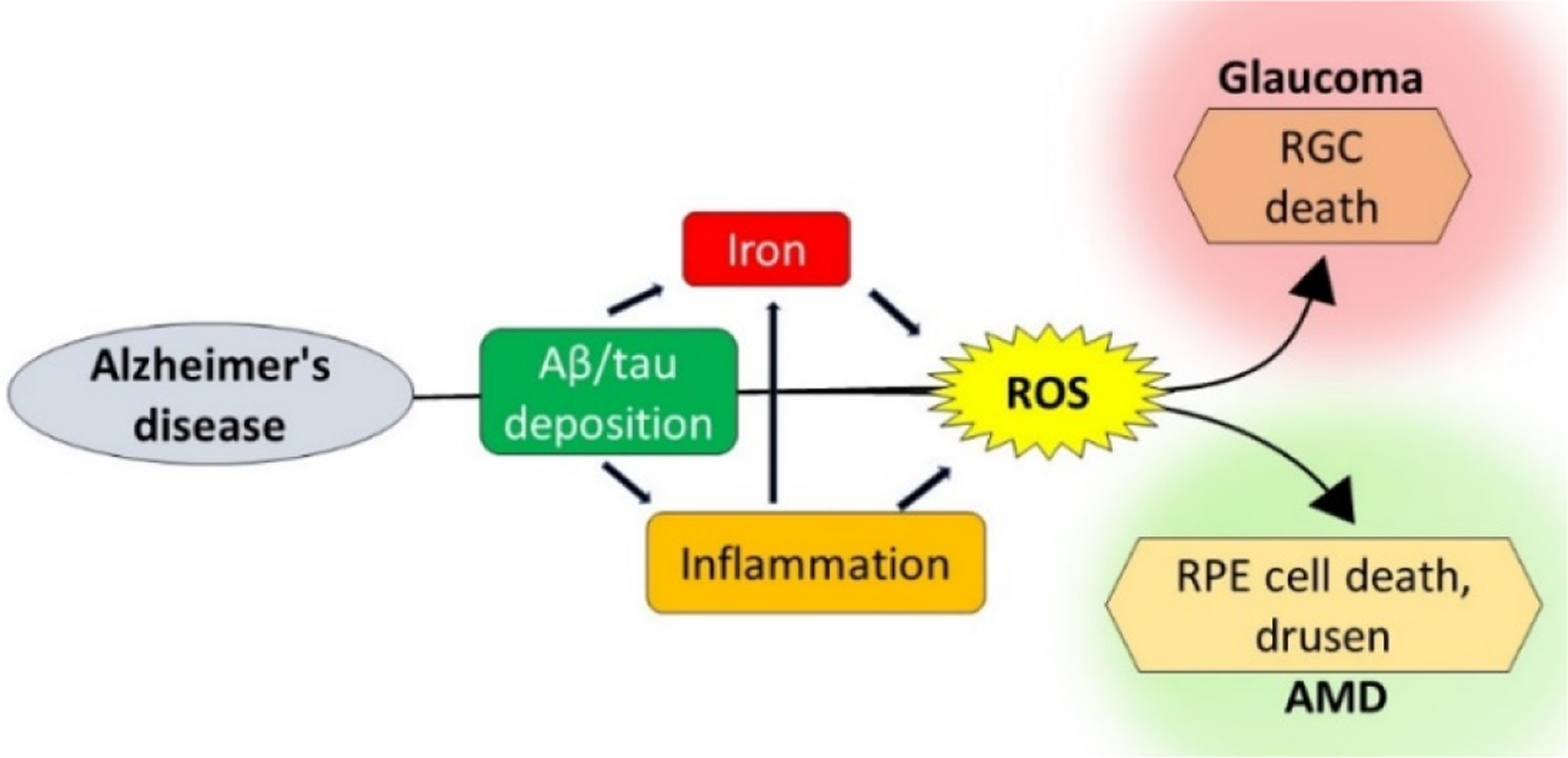
Pathogenic pathways shared by Alzheimer’s disease (AD),AMD, and glaucoma: AD-associated amyloid β (Aβ) and tau deposits lead to inflammation and iron accumulation, increasing ROS. Aβ, hyper-phosphorylated tau (p-tau), inflammation, and ROS together lead to RGC death typical of glaucoma and retinal pigment epithelial (RPE) dysfunction associated with AMD. ROS: reactive oxygen species, RGC: retinal ganglion cells, and AMD: age-related macular degeneration.

Whether these disparate conditions of the brain and the eye share pathogenic pathways remains unclear. The identification of common toxic stimuli could provide an anatomically accessible site for the early diagnosis of AD and the much-needed window for therapeutic management. Here, we review recent information on the pathological processes that are common to AD, glaucoma, and AMD, with emphasis on Aβ, p-tau, chronic inflammation-mediated iron dyshomeostasis, and iron-mediated reactive oxygen species (ROS) (Figure 1). In addition, the role of prion protein (PrP^C^) as a mediator of Aβ toxicity and, paradoxically, as an antioxidant is reviewed.

## 2. Methods

This review is based on the appraisal of existing evidence from multiple systematic reviews linking AD, AMD, and glaucoma. A comprehensive search using the National Library of Medicine, MEDLINE, and Google scholar from 2015 to August 2020, including in vivo, in vitro, and clinical studies, was used to collect and critically appraise the relevant studies.

### 2.1. Aβ and Tau as Mediators of Retinal and Neuronal Degeneration

**Figure 2.**
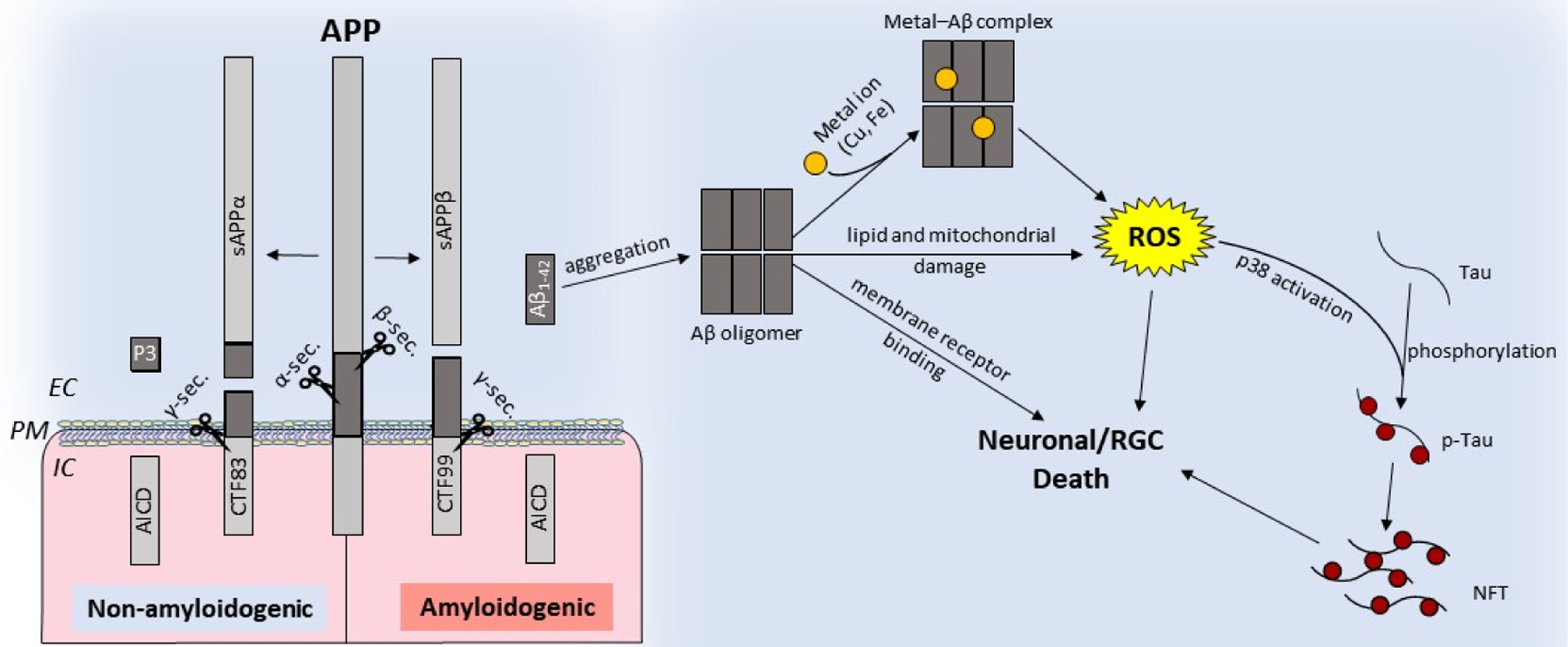
Schematic representation of nonamyloidogenic and amyloidogenic processing of the amyloid precursor protein (APP): nonamyloidogenic processing of APP is mediated by α-secretase followed by γ-secretase, releasing soluble APPα and intracellular fragments AICD and P3. Amyloidogenic processing involves cleavage by β-secretase followed by γ-secretase, which releases toxic Aβ_1–42_ [79,80]. Aβ can oligomerize and form cytotoxic metal-Aβ complexes that generate ROS, disrupt the lipid bilayer, compromise mitochondrial function, or initiate aberrant signaling cascades. Activation of p38 by ROS phosphorylates tau results in NFTs. IC: intracellular, EC: extracellular, NFTs: neurofibrillary tangles, and PM: plasma membrane.

The retina and several other cell types in the eye express amyloid precursor protein (APP), a type I transmembrane glycoprotein, and other proteins implicated in AD. As in the brain, APP undergoes post-translational processing by two mutually exclusive pathways: nonamyloidogenic, and amyloidogenic. The nonamyloidogenic pathway involves the cleavage of APP by α-secretase followed by γ-secretase, and precludes the formation of Aβ. This cleavage occurs at the plasma membrane and is mediated by proteases belonging to the A-disintegrin and metalloprotease (ADAM) family. The resulting amino-terminal fragment APPα is released in the extracellular milieu, and the C-terminal fragment CTF83 is released intracellularly. Subsequent cleavage of CTF83 by γ-secretase releases the APP intracellular domain (AICD) and P3. Processing by the amyloidogenic pathway involves consecutive cleavage of APP by β-secretase and γ-secretase. Cleavage by β-secretase releases soluble APPβ and an intracellular fragment, CTF99. Subsequent cleavage of CTF99 by γ-secretase results in the generation of AICD and Aβ fragments of different lengths. Of these, Aβ_1–42_ is most toxic and accumulates in AD brains as extracellular plaques [1,2,79] (Figure 2). Aβ aggregates bind redox-active metals such as iron and copper, the sources of ROS, and cause mitochondrial damage, leading to neuronal toxicity. Aβ aggregates also cause the hyperphosphorylation and aggregation of tau, compounding the toxicity (Figure 2).

Similar processing of APP occurs in the retina and other cell types in the eye. Soluble APPα, APPβ, and pathogenic Aβ are present in the vitreous and aqueous humor (VH and AH) to varying amounts as in the cerebrospinal fluid (CSF) [61], and pathological deposits of Aβ are prominent in drusen in AMD, and the cause of RGC death and thinning of the RNFL associated with glaucomatous degeneration [81]. These observations are more consistent and prominent in mice models of AD that have been instrumental in gaining insight into the role of Aβ in AD-associated retinal degeneration. The 3xTG-AD, APP-PS1ΔE9, and APPswe/PS1ΔE9 mice that developed Aβ deposits in the brain also show aggregates of Aβ in the retina and thinning of the RNFL [82–84]. Colocalization of Aβ deposits with apoptotic RGCs and axonal degeneration have been reported in several animal models of AD [85,86]. In a rat model of glaucoma, the levels of Aβ_1–42_ in the retina increased with elevated IOP, exposure to light, and ageing, resulting in the apoptotic death of RGCs [87]. These observations were reproduced by intravitreal injection of Aβ_1–42_ [88], and RGCs rescued with agents that reduced Aβ_1–42_ levels or genetic variants that promote nonamyloidogenic processing of APP, indicating a causal relationship.

Deposits of Aβ are not restricted to RGCs. Aβ deposits are present in all layers of the retina, including the ganglion cell layer, nerve fiber layer, photoreceptor layer, and the inner plexiform layer where they promote phosphorylation and accumulation of tau as amorphous deposits and NFTs in the retinal layers and RGCs [81,89,90]. In mice models overexpressing mutant tau, there is a direct correlation between p-tau, Aβ deposits, and RGC death [53]. Interestingly, elevated IOP increases the accumulation of tau and RGC death, and downregulation of tau by short interfering RNA rescues RGCs, confirming tau as the underlying cause [87]. Due to its role in stabilizing microtubules, tau phosphorylation and aggregation interferes with anterograde axonal transport and inhibits mitochondrial transport, resulting in loss of energy and generation of ROS [91]. Additionally, Aβ deposits sequester redox-active metals such as iron and induce toxicity by iron-catalyzed ROS, which causes additional Aβ generation and aggregation, creating a positive feed-forward loop [4,5,92,93]. Moreover, Aβ deposits and intracellular NFTs initiate a cascade of events that activate retinal astrocytes and microglia with the secretion of inflammatory cytokines, including interleukin-1β(IL-1β), IL-6, and tumor necrosis factor α (TNFα) [94,95], which, along with Aβ-generated ROS, create a toxic microenvironment leading to RGC death and thinning of the RNFL.

The triggers that shift the physiological, non-amyloidogenic processing of APP to the amyloidogenic generation of Aβ are not clear, except for the inherited forms of AD. It is also unclear whether the processing of APP in the brain and the eye is altered to the same extent, and the efficiency of Aβ clearance mechanisms in the brain and the eye. In sporadic AD, which forms the bulk of AD cases, the deposition of Aβ and p-tau in RGCs is not a consistent observation [50,52,90]. It is therefore unclear whether the visual symptoms summarized in Table 1 are a direct consequence of degeneration of the visual cortex or result from dysfunction or death of RGCs that receive and transmit the visual stimuli.

The role of Aβ in the pathogenesis of AMD is less clear. Although Aβ deposits have been detected in drusen [93,96,97], the mechanism by which amyloidogenic processing takes precedent over the non-amyloidogenic processing of APP by retinal pigment epithelial (RPE) cells is unclear. It is likely that, during normal ageing, the production and secretion of Aβ_1–42_ by RPE cells increases, which accumulates at the interface of RPE cells and outer segments of the photoreceptors tips and in the subretinal space, where it is engulfed by the microglia [98]. In support of this hypothesis, the overexpression of Aβ in RPE cells induces AMD-like pathology [99] that is exacerbated by bloated microglia that accumulate Aβ deposits and other cellular debris. Together, these changes initiate an inflammatory response and deposition of drusen typical of AMD. A recent meta-analysis of 21 studies showed a significant association between AD and AMD [100], reinforcing the pathogenic role of Aβ in AMD.

Thus, AD and AMD share extracellular Aβ deposits and inflammation as common underlying pathogenic mechanisms. The glaucomatous degeneration of RGCs, in addition, is associated with p-tau. Whether these features suggest a link between the three disorders or are coincidental observations associated with ageing is unclear at present. The association of RNFL thinning with AD, however, suggests a causal relationship. Further exploration is necessary to resolve these questions.

### 2.2. Iron Dyshomeostasis and Reactive Oxygen Species

In addition to deposits of Aβ and p-tau, inflammation is a prominent feature of AD brains [3,101,102]. The chronic activation of resident microglia due to the incomplete degradation of phagocytosed Aβ and neuronal debris releases pro- and anti-inflammatory cytokines, exacerbating the underlying pathology by initiating a cascade of events. Among these, iron dyshomeostasis takes central stage due to its potential to generate ROS and associated toxicity [103]. Although a consistent feature of AD brains, the mechanism of iron accumulation has remained controversial. Recent evidence suggests the cytokine-mediated upregulation of brain hepcidin, the master regulator of iron homeostasis, as the underlying cause [104]. Hepcidin is a peptide hormone secreted mainly by the liver. It maintains systemic iron within a narrow range since iron is essential for vital catalytic reactions, but excess can be highly toxic because of the ease with which it cycles between redox states [105]. This is achieved by modulating the expression of ferroportin (Fpn), the only known iron export protein. Increased iron saturation of serum transferrin upregulates the secretion of liver hepcidin, which binds Fpn on the plasma membrane of cells and causes its internalization and degradation [106]. This decreases both iron uptake from the intestine and release from iron stores, reducing the circulating iron. The opposite scenario takes effect when iron levels are low [107]. Although the brain and the eye are protected from fluctuations in circulating iron by the blood-brain and blood-retinal barriers, respectively, several cell types in the brain, retina, and anterior segment of the eye express hepcidin, suggesting additional regulation of iron exchange locally. In the brain, hepcidin is expressed in the cortex, hippocampus, cerebellum, thalamus, and medulla oblongata [108–110]. In the eye, the synthesis and expression of hepcidin is noted in several cell types in the retina and the anterior segment [111–113].

**Figure 3.**
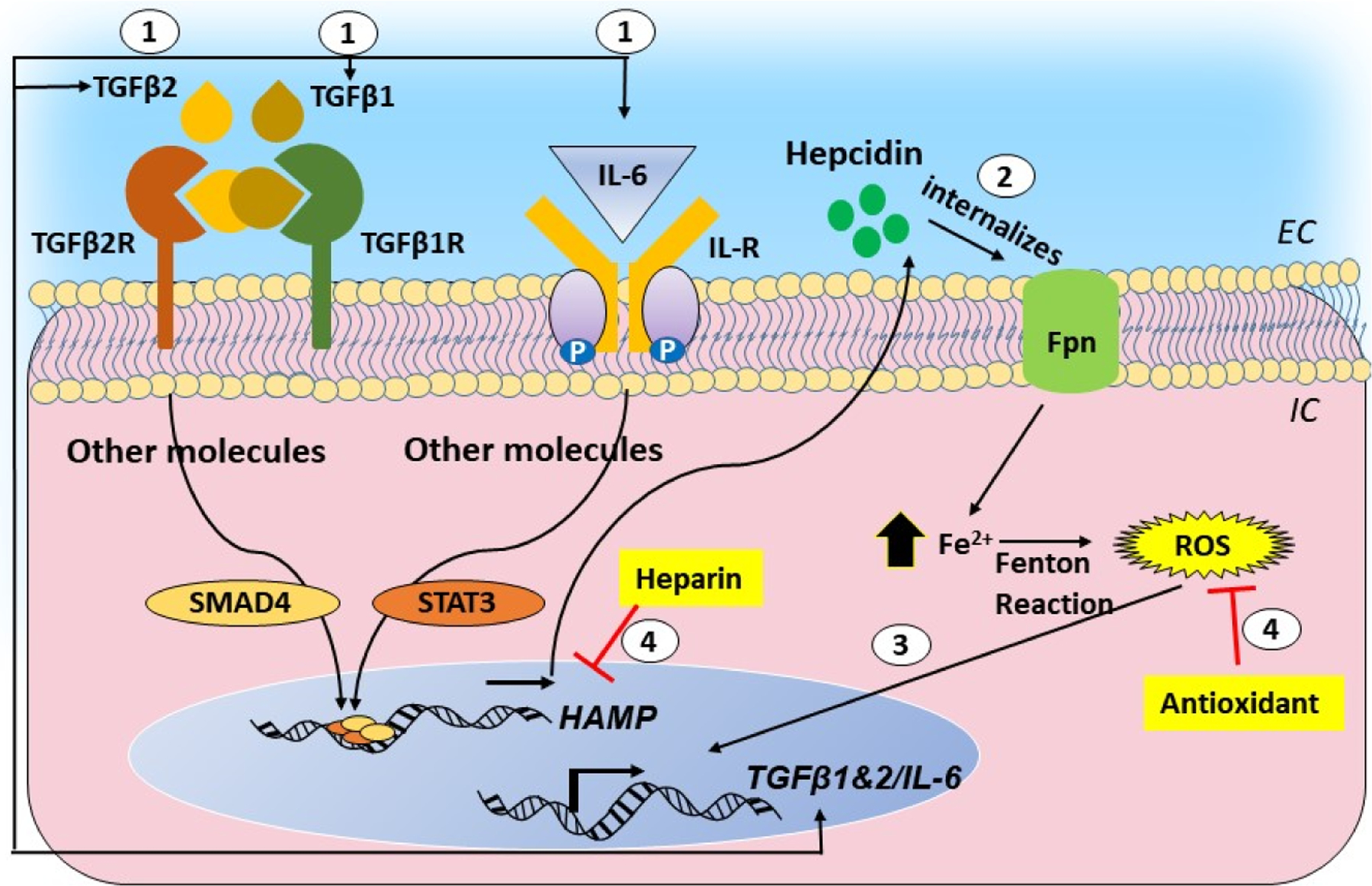
Graphical representation of the cytokine-hepcidin-iron feed-forward loop and its disruption by hepcidin antagonists and antioxidants: (1) transforming growth factor beta 1 and 2 (TGF-β1 and 2) and interleukin (IL)-6 upregulate hepcidin through the SMAD and signal transducer and activator of transcription (STAT)-mediated pathways. (2) Hepcidin causes the downregulation of ferroportin and intracellular accumulation of iron. (3) Iron-catalyzed ROS promotes the transcriptional activation of TGF-β1 and 2 and IL-6, creating a self-sustained feed-forward loop. (4) Hepcidin antagonists and antioxidants disrupt this loop.

Though helpful in maintaining a stringent control of iron, hepcidin is also upregulated by inflammatory cytokines IL-6, IL-1β, and transforming growth factor (TGF) β1 and β2 (Figure 3). The inflammatory signal supersedes the signal from iron, and is the cause of the anemia of chronic inflammation, where iron is sequestered within liver cells and additional uptake is blocked despite low-circulating iron [114]. This raises the possibility of the upregulation of local hepcidin in the brain and the retina of AD cases, which is invariably accompanied by chronic inflammation. The consequent downregulation of Fpn is likely to increase the intracellular iron, creating a toxic environment by increasing ROS. Levels of hepcidin and redox-active iron are increased in AD brain tissue, supporting the above assumption [108]. Unlike systemic circulation, where excess iron is sequestered by the liver to protect vital organs, the brain and the retina lack such protection. The high metabolic rate of the brain and constant exposure of the retina to light provide an optimal milieu for reducing stored, relative stable ferric iron to its redox-active ferrous form, rendering these organs highly susceptible to iron-mediated toxicity by ROS.

As in AD, inflammation accompanies glaucomatous degeneration and AMD, and the release of various cytokines from activated microglia is likely to upregulate hepcidin, leading to an accumulation of iron. Oxidative stress is prominent in glaucoma [115–117], and is exacerbated by the release of TGFβ1 and IL-6, cytokines known to trigger the upregulation of hepcidin. In a recent report, the upregulation of hepcidin in TM cells by TGFβ2 initiated a positive feed-forward loop between TGFβ2, hepcidin, and iron fueled by ROS. Disruption of this loop with hepcidin antagonists and antioxidants reduced the iron accumulation and ROS, suggesting a prominent role of ROS in primary open-angle glaucoma [111,118] (Figure 3). In addition, significant protection of RGCs is achieved by chelating iron, reinforcing the toxic role of iron in glaucomatous degeneration [119].

The toxic role of iron is better understood in AMD, where intravenous iron and hereditary diseases associated with systemic iron overload, such as aceruloplasminemia, pantothene kinase deficiency, and Friedrich’s ataxia, show AMD-like retinal degeneration. Mice lacking ceruloplasmin and hephaestin, ferroxidases necessary for iron export, reproduce several features of AMD, and levels of transferrin, an iron uptake protein, are elevated in the retina and AH of AMD cases [118,120–122]. Elevated serum iron is also associated with retinal degeneration with AMD-like pathology, indicating a direct role of iron in retinal degeneration.

### 2.3. Clinically Relevant Therapeutic Options

Various anti-Aβ therapies have been tried for AD, AMD, and AD-associated glaucomatous degeneration, and have been discussed in excellent reviews [123]. Here, we describe recent developments in therapeutic options that reduce iron-catalyzed ROS and oxidative stress by iron chelators, hepcidin antagonists, and Fpn-stabilizing agents.

Partial success in ameliorating the symptoms of AD, glaucoma, and AMD with iron chelators re-enforces the central role of iron-mediated ROS in AD pathogenesis [124,125]. For example, iron chelators have been used to ameliorate the symptoms of AD with partial success [126]. Recently, deferiprone (DFP), a potent iron chelator, has been shown to rescue RGCs and glaucomatous degeneration in a mouse model of glaucoma [127,128]. In Abca4^−/−^ mice, a widely used model of retinal degeneration, deferiprone reduced the oxidation-driven degradation of vital bisretinoids such as A2E [129]. In mice lacking ceruloplamin and hepaestin, which accumulate iron in the retina, salicylaldehyde isonicotinoyl hydrazine (SIH), a potent iron chelator, rescued the phenotype. SIH also provided efficient protection against H_2_O_2_-induced cytotoxicity in an in vitro model of AMD relative to other iron chelators such as deferoxamine, which had visual side effects.

Hepcidin antagonists are another class of small molecular weight compounds that reduce hepcidin-mediated accumulation of iron. These are likely to prove more beneficial in reducing the iron accumulation associated with chronic inflammation [130]. Some of the hepcidin antagonists are anti-inflammatory compounds, such as IL-1 receptor inhibitors, anti-IL-6 monoclonal antibody, and TNF-α blockers [131,132]. These agents, in addition to reducing the hepcidin-mediated accumulation of iron, are likely to reduce inflammation as well, adding to their therapeutic potential. In an in vitro model of glaucoma, the cytokine-mediated upregulation of hepcidin was disrupted with heparin, a hepcidin antagonist, and N-acetyl L carnosine (NAC), an antioxidant [111], suggesting that such agents are useful in decreasing the iron-catalyzed ROS and may prove useful in rescuing neurons and RGCs from ROS-mediated toxicity and death. Inflammatory changes accompany glaucomatous degeneration [131], and the use of anti-inflammatory compounds, including nonsteroidal anti-inflammatory drugs (NSAID) such as pranoprofen, have been useful in decreasing the IOP and rescue RGCs [133]. Several such compounds are undergoing clinical trials for systemic disorders of iron overload [134], and could be modified for use in the brain and the eye.

### 2.4. The Paradoxical Role of Prion Protein

**Figure 4.**
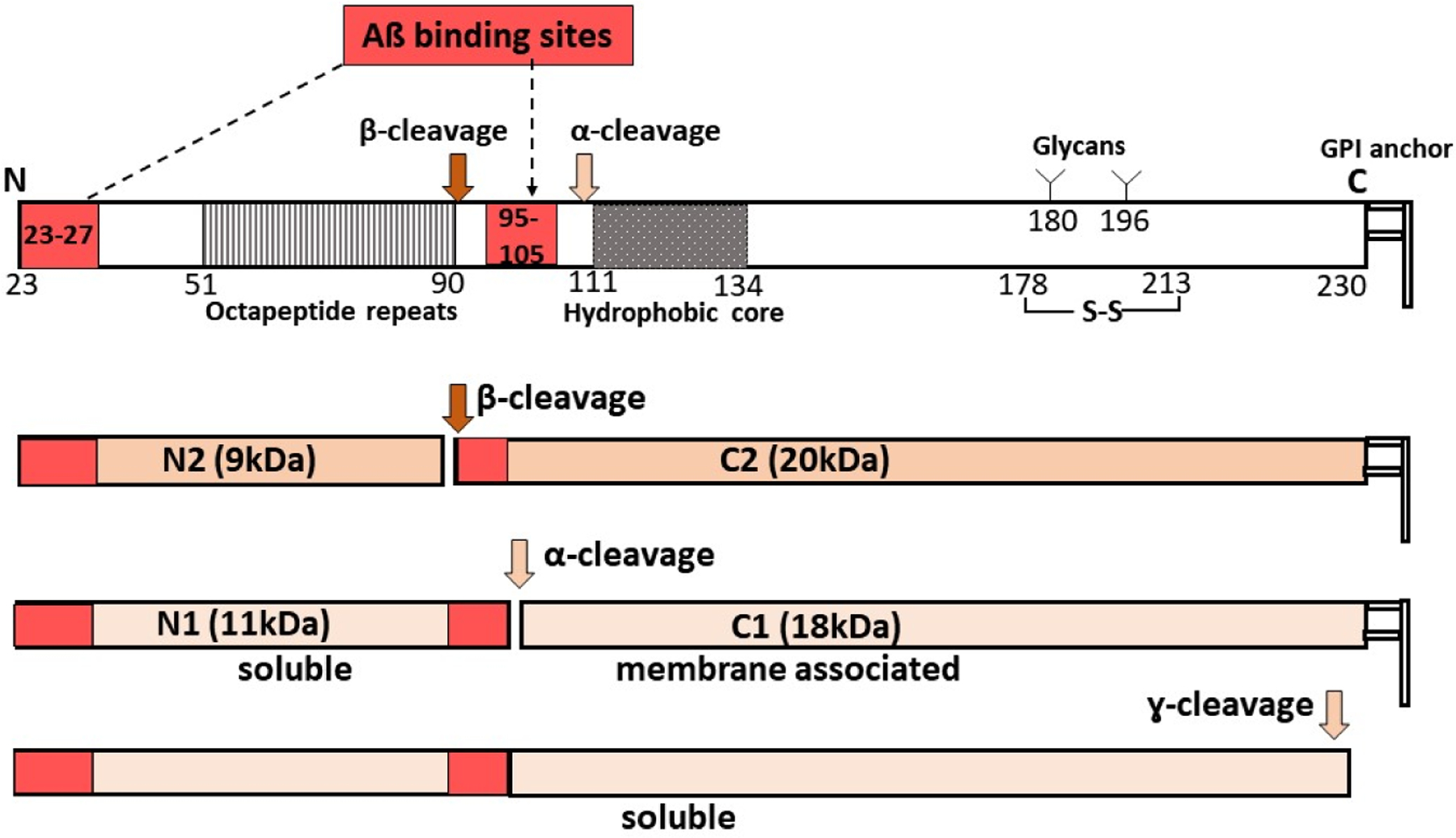
Processing of prion protein (PrP^C^) and Aβ binding: PrP^C^ undergoes α-, β-, or γ-cleavage, releasing extracellular N-terminal fragments N1 (α-cleavage), N2 (β-cleavage), or soluble PrP^C^. Full-length PrP^C^ has two Aβ binding sites. N2 has one, and N1 and soluble PrP^C^ have two Aβ binding sites that could sequester soluble Aβ. The truncated C2 and C1 are attached to the plasma membrane, and the presence of one Aβ-binding site on C2 is likely to transmit the toxic signal of Aβ.

The prion protein (PrP^C^) is a ubiquitously expressed protein mainly known for its role as the substrate for PrP-scrapie (PrP^Sc^), the principal pathogenic agent responsible for all prion disorders [135,136]. Besides its pathological role, several physiological functions are attributed to PrP^C^, including protection from oxidative stress, iron uptake, and in regulating the levels of TNFα-cleaving enzyme (TACE) [137–141]. The significance of PrP^C^ in the pathogenesis of AD stems from its role as a receptor for Aβ oligomers [142,143]. Aβ_1–42_ specifically binds PrP^C^ with high affinity in a saturable and reversible manner, and mediates biologically relevant downstream intracellular signaling events including the loss of synaptic function, impaired memory and cognition, and other functional deficits associated with AD [144,145]. Two binding sites of Aβ have been identified on PrP^C^: residues 95–105 and residues 23–27 [143] (Figure 4). Deletion of 95–105 residues or blocking this site with antibodies interferes with the Aβ-PrP^C^ interaction, rescuing the toxicity of Aβ. Additionally, the binding of Aβ oligomers to PrP activates the tyrosine kinase Fyn pathway, leading to synaptic dysfunction and loss [146].

It is interesting to note that, under steady-state conditions, ~65–80% of PrP^C^ on neuronal cells undergoes physiological cleavage at residues 111/112, releasing the N-terminal soluble fragment N1 in the extracellular milieu and C-terminal fragment C1 attached to the plasma membrane. This is also referred to as α-cleavage and occurs in an endocytic compartment during the recycling of PrP^C^ from the plasma membrane. This cleavage is mediated by ADAM17 [147]. Since C1 lacks the primary Aβ-binding site, this cleavage is believed to protect the neurons from Aβ-induced toxicity. The released N1 fragment, on the other hand, is likely to bind and sequester Aβ in the extracellular milieu and protect the cells from toxicity. However, in the retina, ~90% of PrP^C^ is cleaved around residue 90, leaving the primary Aβ-binding site intact on the C-terminal fragment or C2. The released N-terminal fragment N2 contains the secondary Aβ-binding site spanning residues 23–27. Also called the β-cleavage of PrP^C^, this event is mediated mainly by oxidative stress and is believed to protect the cells from free radical damage [148] (Figure 4). It is likely that the presence of β-cleaved PrP^C^ in retinal cells and other cell types in the eye is due to the constant exposure to light, a source of oxidative stress, or reduced expression of ADAM17. This enzyme is also responsible for the α-cleavage of APP that precludes its amyloidogenic processing [149]. The relative paucity of Aβ deposits in confirmed cases of AD makes it unlikely that the preferred β-cleavage of PrP^C^ is due to the lack of ADAM17. Further exploration is necessary to resolve this question. Nevertheless, the genetic deletion of PrP^C^ reduces Aβ binding by ~50%, implicating PrP^C^ in Aβ-mediated toxicity [150]. It is likely that additional cell-surface proteins participate in this process, or distinct oligomeric species of Aβ bind PrP^C^.

Paradoxically, PrP^C^ protects the retina from light-induced oxidative damage and stabilizes ECM proteins by interacting with β1 integrin [151,152]. The down-regulation of PrP^C^ activates the Ras homolog gene family member A (RhoA)-associated coiled-coil containing kinase (ROCK) pathway, resulting in the overactivation of ROCK and signaling through the LIMK-cofilin pathway [153,154]. The net result is a shift from cell-cell interactions to cell-substrate interactions. In the eye, this results in increased stiffness of the ECM, resistance to aqueous outflow, and elevated IOP, resulting in RGC death. In the brain, PrP^C^ protects the cells from free radicals, with the result that mice lacking PrP^C^ are more susceptible to intracellular ROS [155]. It is likely that chelation of redox-active metals such as copper and iron by the N-terminal octapeptide repeats provide protection from metal-catalyzed ROS [156–159]. At the same time, this region is involved in copper and iron transport [141,160,161], suggesting that PrP^C^ maintains an adequate intracellular concentration of these metals by shedding off the octapeptide repeat region, a hypothesis that requires further testing.

## 3. Conclusions

In this review, common pathogenic mechanisms and differences between AD and age-related and glaucomatous degeneration are highlighted, with emphasis on Aβ, p-tau, and inflammation-mediated iron dyshomeostasis as the common underlying cause. In AD, the incomplete degradation of Aβ and p-tau by microglial cells is likely to trigger the release of cytokines, which upregulate local hepcidin and iron accumulation in the brain. In glaucomatous degeneration, inflammation initiated by Aβ and p-tau deposits due to amyloidogenic processing of APP in the retina and RGCs is likely to increase cytokine levels, upregulating retinal hepcidin and the accumulation of iron. The elevation of IOP has a similar effect, blurring the distinction between AD-associated glaucoma and RGC death due to elevated IOP. AMD is associated with inflammation, the accumulation of iron, and Aβ deposits in drusen, suggesting a strong correlation with AD. However, both AD and AMD are diseases of advanced age, and it is difficult to attribute these changes to AD per se. The increased synthesis of hepcidin due to inflammation is common among the three conditions and offers an untapped opportunity to reduce iron-catalyzed ROS with hepcidin antagonists. Iron chelators have proved beneficial in treating AD, AMD, and glaucoma. Hepcidin antagonists are likely to be more effective if delivered locally in the brain or the eye in optimal amounts. Several such compounds are undergoing clinical trials for systemic disorders of iron accumulation, and could be modified for use in the brain and the eye.

PrP^C^, though apparently disconnected, is involved in all three disorders. First, it links neuronal and RGC death by serving as a mediator of Aβ toxicity. Second, PrP^C^ transports iron across biological membranes, contributing to the accumulation of iron. Third, the unusual processing of PrP^C^ in the retina at the β-site suggests that PrP^C^ offers protection from increased oxidative stress probably from constant exposure to light. Paradoxically, the absence of PrP^C^ increases the IOP by altering the characteristics of the ECM, which is likely to promote the accumulation and oligomerization of p-tau in RGCs. Additional studies are required to clarify the specific role of PrP^C^ in the eye.

## Figures and Tables

**Table 1.** Ocular symptoms associated with Alzheimer’s disease (AD).

Tissue	Symptoms	Contradictory Findings
**Clinical Manifestations**		
Visual Dysfunction	Impaired contrast sensitivity [16–20], color vision [16,17,19,20], visual acuity [17,19], and visual integration [17]. Macular thinning [16,17], visuospatial deficits [18–20], visuomotor impairment [21–24], visual field loss [25]	
Cornea	Increased corneal sensitivity [26], corneal thinning [27,28]	
Retina	Reduced retinal nerve fiber layer thickness in superior and inferior quadrants [13,16,29,30], retinal astrogliosis [31], reduction of retinal ganglion cells [13,32] most notably in fovea [32]	
Pupil	Increased pupil diameter during cognitive effort [33], slowed pupillary responses to light and target detection task [34], exaggerated pupil response with dilute tropicamide [35,36], smaller baseline pupil size [37]	
Lens	Equatorial supranuclear cataracts [38,39]	Opacity not related to Alzheimer’s disease [40,41]
Optic Nerve	Axonal degeneration [42,43], thinner lamina cribrosa [28]	No axonal damage [44]
Intraocular pressure	Increased intraocular pressure in Alzheimer’s disease patients [45,46]	
**Pathological Changes**		
Cornea	Fibroblasts, epithelium express amyloid precursor protein and amyloid β [47], A disintegrin and metalloproteinase domain-containing protein ADAM-10 and beta-site amyloid precursor protein cleaving enzyme 1 increased in fibroblasts [47], decreased microvilli and altered morphology in corneal epithelia [27], several-fold increase in amyloid precursor protein expression, amyloid β deposition, and increased apoptosis in corneal epithelia [27]	
Retina	Reduced oxygen metabolism [48,49], amyloid β plaques [50,51], accumulation of phospho-tau [52,53], amyloid β deposition in retinal microvasculature and pericytes [54], compromised blood-retinal barrier [54], retinal vasculature abnormalities [55,56], increased retinal vascular amyloid β_40_ and amyloid β_42_, decreased vascular platelet-derived growth factor receptor β, and decreased vascular low-density lipoprotein −1 [54]	
Lens	Increased amyloid β aggregation in lens [38,57], presenilin expression in lens [58]	No aggregation of amyloid β [40,41]
Aqueous Humor	Increased levels of amyloid β [59,60]	
Vitreous Humor	Presence of Alzheimer’s disease -associated proteins [59,61]	
Choroid	Thinning of choroid [62,63]	
Optic Nerve	Tau deposition [20]	

## Abbreviations

AD: Alzheimer’s Disease
ADAM: A-disintegrin and metalloprotease
AH: Aqueous Humor
AMD: Age related macular degeneration
APP: Amyloid Precursor Protein
Aβ: Amyloid β
CSF: Cerebrospinal Fluid
DFP: Deferiprone
ECM: Extracellular Matrix
ERG: Electroretinography
Fpn: Ferroportin
IL: Interleukin
IOP: Intraocular Pressure
NFT: Neurofibrillary Tangles
NOS: Nitric Oxide Synthase
OCT: Optical Coherence Tomography
PrP^C^: Prion Protein
PrP^Sc^: Prion Protein Scrapie isoform
p-tau: phosphorylated tau
RGC: Retinal Ganglion Cells
RhoA: Ras homolog gene family member A
RNFL: Retinal Nerve Fiber Layer
ROCK: Rho-associated protein kinase
ROS: Reactive oxygen species
SIH: Salicylaldehyde Isonicotinoyl Hydrazine
TACE: Tumor Necrosis Factor α cleaving enzyme
TGFβ2: Transforming growth factor beta-2
TM: Trabecular meshwork
TNFα: Tumor Necrosis Factor α
t-tau: Total tau
VH: Vitreous Humor
